# Notes and News

**Published:** 1902-11-22

**Authors:** 


### NOTES AND NEWS.

There are 31 nominations for 21 places on the new board of the Manchester Infirmary. Amongst the candidates are three ladies.

Messrs. Cook and Sons have arranged special terms for members of the Egyptian Medical Congress for the journey from London to Cairo and for tours on the Nile.

Mr. H. J. Tresidder having resigned the secretaryship of the National Orthopædic Hospital for the Deformed, 234 Great Portland Street, W., after more than nine years' service, owing to ill-health, Mr. Keith Anstruther has been appointed by the committee of management as secretary.

The Local Government Board has added another to a useful series of circulars respecting vaccination administration and the prevention of small-pox, intended to facilitate the full and particular classification of cases. The form of "register" drawn up by the Board's medical officer, Mr. W. H. Power, with a "vaccination bed-card" for the accurate notation of the previous vaccination history of each small-pox patient, will certainly be welcomed in the hospitals of the Metropolitan Asylums Board.

On Wednesday and Thursday afternoons, December 3rd and 4th, there will be a sale of the goods which were left over from the Coronation Bazaar at the Hospital for Sick Children, Great Ormond Street. Princess Henry of Battenberg will perform the opening ceremony on Wednesday at 3 p.m., and on Thursday the Bazaar will open at 2 p.m. Two stalls will be presided over by the matron and nurses, and others will be furnished by members of "The Children's Salon," who are collecting money to endow a cot at the Hospital.

The foundation stone of a new cottage hospital has been laid at Caterham.

A new sanatorium for consumptives has been opened at Knightwick, Worcestershire, for the poor of that county. It will contain 16 patients, for the maintenance of whom £800 per annum for five years has already been guaranteed. Mr. Dangerfield, of Bilston, has lent a mansion for use during five years, and shelters have been added to it.

Accounts are at variance as to the extent of the outbreak of cholera in Palestine, but it appears certain that it is present in Jaffa, Gaza Lydd, Tiberias, Hebron, and Nablous. One case is reported at Jerusalem. Active measures are being taken to check the spread of the disease. Cholera, too, is still rife in Egypt, and is likely to keep English visitors in Europe during the winter and spring this season.

The Northampton Infirmary is to be congratulated on the interest awakened on its behalf, which was strikingly demonstrated at the special meeting of the Governors held recently, when the Hospital Week Fund, the Northampton Infirmary Sports Committee, and the Sunday Fund sent representatives to the hospital for the purpose of presenting cheques, the result of collections on behalf of the institution. The Hospital Week Fund, as now worked, is a new organisation, and the handsome donation of £1,000 made on this occasion was the result of a collection during six months only from the various firms in Northampton. Mr. Manfield, who presented the cheque and spoke on behalf of the Fund, said that new firms were continuously joining the movement, so that an assured and substantial income may be looked for by the infirmary. As the Sports Committee and Sunday Fund are also arousing interest and securing more pecuniary aid for the institution, it is to be hoped that with a new building the Northampton Infirmary will become prosperous and efficient in the near future.

This is the age of automatic action, the day of penny-in-the-slot control, and no doubt such régime has its advantages. At the Brewers' Exhibition at the Agricultural Hall, automatic machines for the distribution of "drinks" were exhibited. The machines, we understand, can be made to serve any kind of liquor, in any pre-arranged quantities, and at any desired price. Customers have only to put the necessary number of coins in the slot, turn the tap, and the exact measured quantity of liquid is at once automatically delivered in the glass, the whole operation taking only a few seconds. If such a mechanism becomes popular, we shall have to face a new phase of the "Drink Question," and where "intoxicants" are dispensed by "automatic action" it is very probable that we shall be compelled to seek fresh legislative restrictions. We trust that all interested in the spread of temperance and the discouragement of drunkenness will see that this new invention is made good use of in furthering the distribution of pleasant and harmless beverages, and that its employment for the disposal of alcoholic drinks is discouraged.

